# PROTACs for BRDs proteins in cancer therapy: a review

**DOI:** 10.1080/14756366.2022.2081164

**Published:** 2022-06-14

**Authors:** Chao Wang, Yujing Zhang, Shanbo Yang, Wujun Chen, Dongming Xing

**Affiliations:** a The Affiliated Hospital of Qingdao University, Qingdao Cancer Institute, Qingdao University, Qingdao, PR China; b The Affiliated Cardiovascular Hospital of Qingdao University, Qingdao University, Qingdao, PR China; c School of Pharmacy, Qingdao University, Qingdao, PR China; d School of Life Sciences, Tsinghua University, Beijing, PR China

**Keywords:** BRDs proteins, inhibitor, PROTACs, degradation, promising treatment

## Abstract

BRDs proteins that recognise chromatin acetylation regulate gene expression, are epigenetic readers and master transcription coactivators. BRDs proteins are now emerging as targets for new therapeutic development. Blocking the function of any of BRDs proteins can be a control agent for diseases, such as cancer. Traditional drugs like enzyme inhibitors and protein–protein inhibitors have many limitations. The therapeutic efficacy of them remains to be proven. Recently, Proteolysis-Targeting Chimaeras (PROTACs) have become an advanced tool in therapeutic intervention as they remove disease-causing proteins. Extremely potent and efficacious small-molecule PROTACs of the BRDs proteins, based on available, potent, and selective BRDs inhibitors, have been reported. This review presents a comprehensive overview of the development of PROTACs for BRDs proteins regulation in cancer, and the chances and challenges associated with this area are also highlighted.

## 1. Introduction

**Figure 1.**
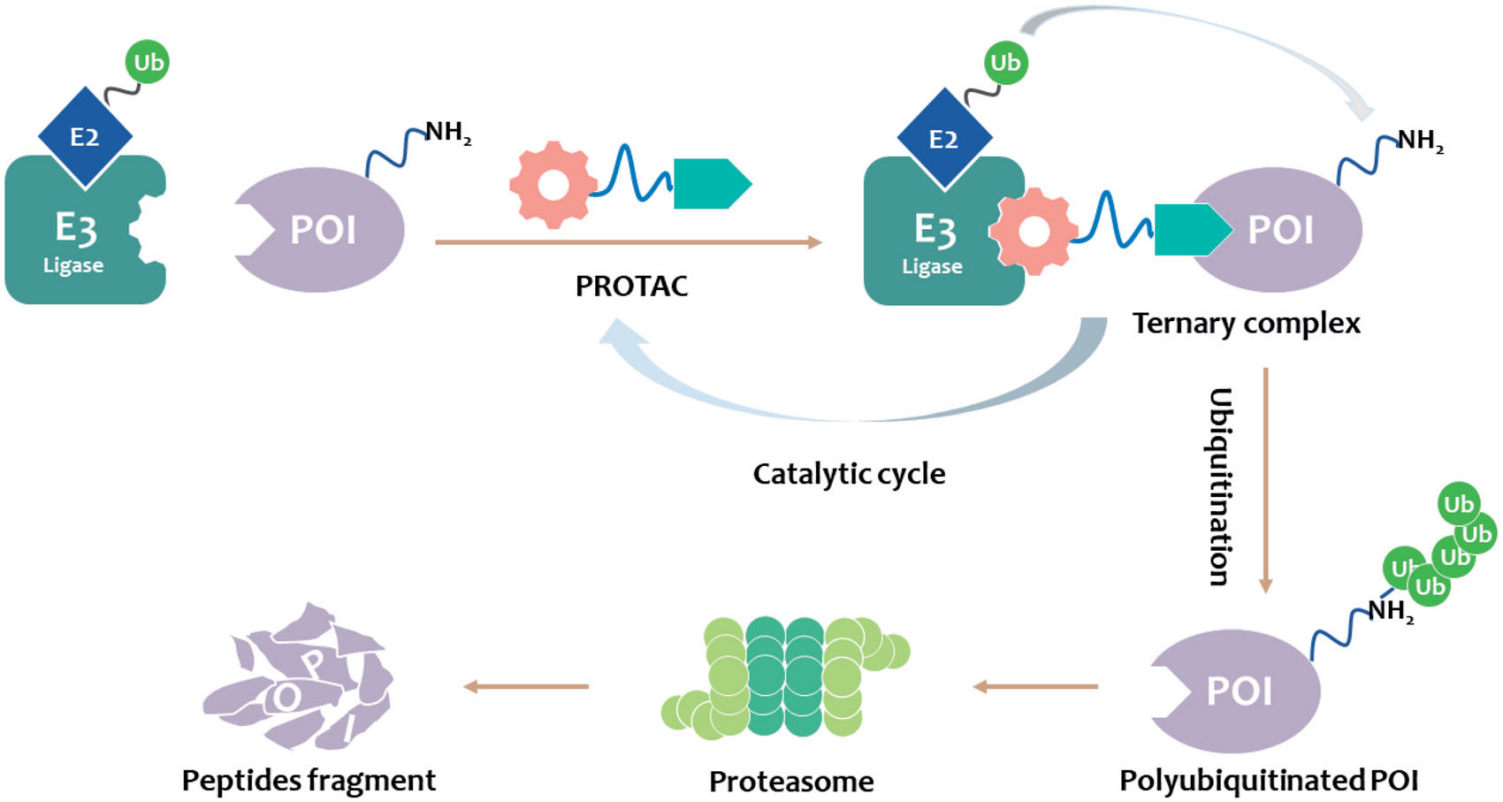
PROTAC-mediated degradation of target proteins through the UPS.

In the past 20 years, a novel strategy that targets disease-related proteins for degradation has gained tremendous attention. Proteolysis targeting chimerics (PROTACs), also known as bivalent chemical protein degraders, are heterobifunctional molecules that degrade specific endogenous proteins through the E3 ubiquitin ligase pathway. It structurally connects the protein of interest (POI)-binding ligand and the E3 ubiquitin ligase (E3) ligand through an appropriate linker^1–7^. The main mechanism of PROTACs technology is to use UPS to degrade the proteins of interest (POI). The E3 ligase ligand of PROTAC can hijack the E3 ligase and label the POI with ubiquitin. In this process, PROTAC itself is not degraded, instead, it is recycled to promote ubiquitination and degradation of other target proteins (Figure 1)^2^^,^^5^^,^^8^^,^^9^^,^^10^. This catalytic, event-driven modality^11^ operates in contrast to the function of conventional inhibitors, in which sequential target binding is necessary to stimulate the desired effect. For standard occupancy-driven typical small-molecule drugs, binding affinity is necessary for their efficacy. In contrast, PROTACs induce degradation of POI by ubiquitin-proteasome system (UPS), an event-driven modality that can be used to overcome common drawbacks of traditional occupancy-driven small-molecule drugs^11–13^. The potential advantages of PROTAC technology may compensate for the shortcomings of traditional drug therapy, which promotes its rapid development.

**Figure 2.**
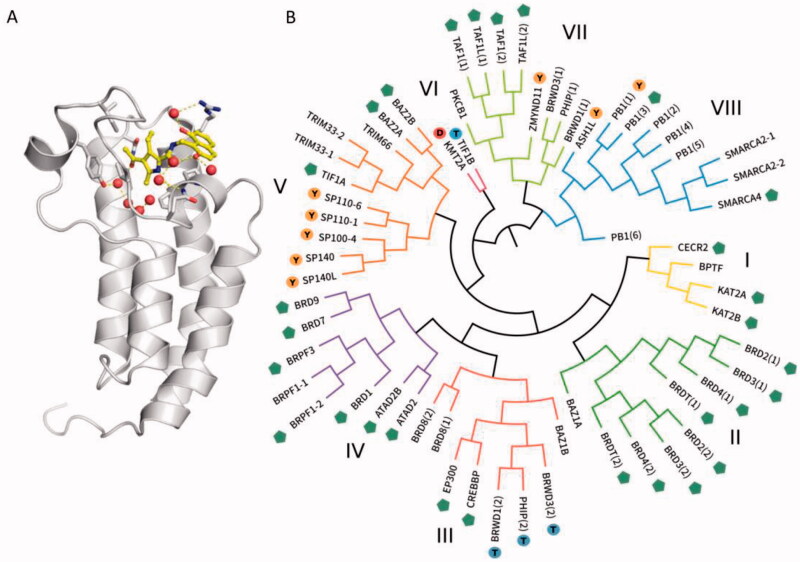
(A) Structure of the human BRDs of transcriptional coactivator CREB-binding protein CREBBP (PDB: 5NU3). The KAc binding site is blocked by the inhibitor XDM-CBP (shown in yellow)^27^^,^^58^. (B) Phylogenetic tree of the human BRDs family. The green branches represent proteins such as BRD2, BRD3, BRD4, etc. The purple branches represent proteins such as BRD1, BRD7, and BRD9^59^.

During carcinogenesis, extensive epigenetic modifications occur, including aberrant acetylation, and methylation patterns. Acetylation of histone lysine residues is one of the most essential post-translational processes that regulates chromatin structure so that it is accessible to DNA and RNA polymerases as well as transcription factors. These alterations result in dysregulated gene expression and abnormal cell proliferation^14^. A key modular domain recognising the acetyl-lysines in a histone is the bromodomain (BRD)^15^. To date, 41 BRDs proteins have been identified^16^ and 30 of these have been shown to bind to the acetyl lysine coded in different segments of the histone tails (Figure 2)^15^. BRDs proteins are known to play a role in cancer and a number of other human diseases^17–21^. In other cases, BRDs either acquire mutations in diseases^22^^,^^23^ or participate directly in the aberrant epigenetic regulation by collaborating with other dysfunctional chromatin modifying enzymes and transcription factors^24^. The bromo- and extra-terminal (BET) family of proteins, including the ubiquitously expressed BRD2, BRD3, BRD4, and the testis-specific BRDT, recruit transcriptional regulatory complexes to acetylated chromatin thereby controlling specific networks of genes involved in cellular proliferation and cell cycle progression^25^. Alterations in regulation of activities from BET protein, especially BRD4, have been greatly allied with cancer and inflammatory diseases. BRD9 is the BRD-containing subunit of the BAF (BRG-/BRM-associated factor) and its close homolog BRD7 is the subunit of PBAF (polybromo-associated BAF)^26^^,^^27^. BAF and PBAF are two variants of the SWI/SNF complex, which regulate gene expression, DNA replication, and DNA repair^28^^,^^29^. Overexpression of BRD7/9 can lead to cancers development. These make BRDs proteins appealing drug targets.

**Figure 3.**
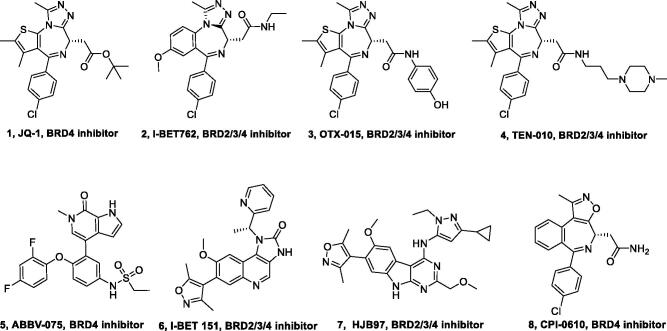
Representative BRDs inhibitors.

The catalytic role of BRDs proteins in transcription led to the development of small-molecule inhibitors of BRDs^30^. JQ-1 is the first and most thoroughly studied BRDs inhibitor^31^. The chemical structure was inspired by a patent of similar BET inhibitors by Mitsubishi Tanabe Pharma [WO/2009/084693] and is related to benzodiazepines. The interference of JQ-1 induces immediate apoptosis in BRD4-dependent human carcinoma cells and reduces tumour growth of NUT midline carcinoma (NMC) in patient-derived xenograft models^30^. With the BRDs inhibitor JQ-1, a remarkable success story of BRD4 as a novel drug target has been set off that yielded many BRDs inhibitors (such as I-BET 762^32^, OTX-015^33^, TEN-010^34^, ABBV-075^35^, and I-BET 151^36^) that are now in clinical trials (Figure 3). Early phase clinical trials results however, show that BRDs inhibitors achieve only modest clinical activity as single agents in patients with advanced cancer^27^^,^^33^. Because BRDs proteins contain multiple functional domains including an extra terminal domain that interacts with transcription factors responsible for ribosomal RNA production, small-molecule BRDs inhibitors may only block their chromatin binding functions. Consequently, new strategies, such as PROTACs could be more much effective for the treatment of human diseases in which BRDs proteins play a key role^37^. Herein, we provide a review of the development of PROTACs for BRDs proteins regulation in cancer, and the chances and challenges associated with this area are also highlighted.

## 2. BRDs PROTACs

### 2.1. CRBN-based PROTACs

#### 2.1.1. Targeting BRD4

**Figure 4.**
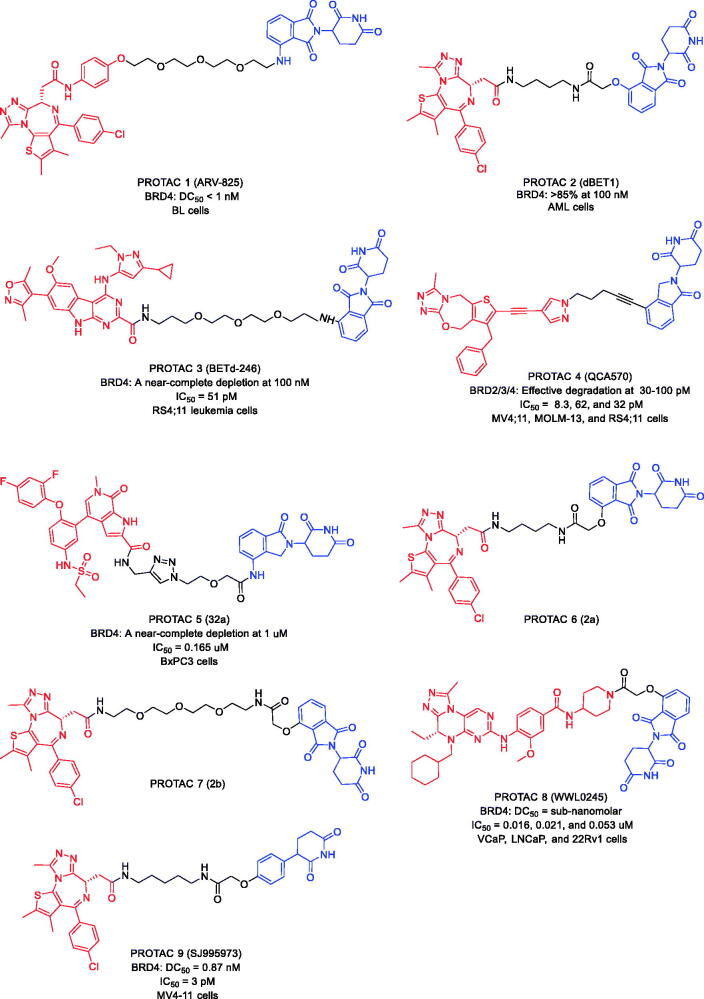
Representative PROTACs targeting BRD4.

In 2015, Lu et al. developed the BRD4 PROTAC (PROTAC 1, Figure 4) by combining BRD4 inhibitor OTX015 and cereblon (CRBN) ligand pomalidomide with an optimised PEG linker^38^. PROTAC 1 induced degradation of BRD4 at nanomole concentration in Burkitt’s lymphoma (BL) cells, with a DC_50_ value below 1 nM. Compared to the high concentration of OTX015, PROTAC 1 showed a more significant effect on c-MYC and downstream cell proliferation as well as apoptosis induction in BL cells.

In the same year, Winter et al. described another well-known BRD4 PROTAC, PROTAC 2 (Figure 4) by conjugating CRBN ligand phthalimide and BRD4 inhibitor JQ-1^39^. In acute myeloid leukaemia (AML) cells, BRD4 was efficiently degraded after treatment with PROTAC 2 at a concentration of 100 nM. In addition, the mice tolerated PROTAC 2 treatment well for 2 weeks without affecting their body weight, number of white blood cells haematocrit values, or platelet counts. There was no obvious toxicity during the treatment with PROTAC 2. Their findings provide strong evidence that BRD4 PROTACs offer a better and more effective strategy than the traditional small molecule inhibitor JQ-1 in targeting BRD4.

In 2017, Bai et al. combined azacarbazole-based BRD4 inhibitor HJB97 with CRBN ligand thalidomide and successfully synthesised a new BRD4 PROTAC (PROTAC 3, Figure 4)^40^. PROTAC 3 could degrade BRD4 protein at the concentrations of 0.1–0.3 nM in the RS4;11 leukaemia cells with the half maximal inhibitory concentration (IC_50_) value of 51 pM. Besides, PROTAC 3 induced regression of RS4;11 xenograft tumours *in vivo*. Compared to conventional BRD4 inhibitors, PROTAC 3 showed lower toxicity. Thus, PROTAC 3 is an effective BRD4 degrader.

In 2018, Qin et al. also disclosed a new BRD4 PROTAC (PROTAC 4, Figure 4), derived from BRD4 inhibitor QCA-276 and CRBN ligand lenalidomide^41^. PROTAC 4 was the most potent BRD4 degrader reported to date, potently degrading BRD4 at picomolar concentrations. Moreover, PROTAC 4 significantly inhibited the growth of MV-4–11, MOLM-13, and RS4;11 cell growth with IC_50_ values of 8.3, 62, and 32 pM, respectively.

In 2020, Zhang et al. reported some novelty BRD4 PROTACs with BRD4 inhibitor ABBV-075 and CRBN ligand lenalidomide^42^. The novel BRD4 degraders showed relatively strong potency against BRD4 BD1 with IC_50_ at nanomolar concentrations. The anti-proliferative activity of PROTAC 5 (Figure 4) against BxPC3 cell lines (IC_50_ = 0.165 μM) was increased approximately 7-fold compared to ABBV-075. In addition, PROTAC 5 effectively degraded BRD4 and inhibited c-Myc expression in a time-dependent manner in BxPC3 cell lines.

In 2021, Bemis et al. demonstrated a parallel, one-pot method for the assembly of PROTACs (PROTAC 6 and PROTAC 7, Figure 4) utilising activated esters generated *in situ*, and traceless Staudinger ligation chemistry^43^. The method described allows for rapid structure-activity relationship studies of PROTACs linker variants. Two previously studied systems, CRBN, and BRD4 degraders, are examined as test cases for the synthetic method. The two related strategies to assemble PROTACs linker variants discussed can accommodate the chromatographic separations capabilities of labs of many sizes and incorporates commercially available degrader building blocks, thereby easing synthetic entry into PROTACs chemical space.

In the same year, Hu et al. designed and synthesised a series of PROTACs based on their recently reported dual BET/PLK1 inhibitor WNY0824, which led to the discovery of an isoform-selective and potent BRD4 PROTAC (PROTAC 8, Figure 4)^44^. PROTAC 8 exhibited excellent selective cytotoxicity in the BET inhibitors sensitive cancer cell lines, including AR-positive prostate cancer cell lines. It could also efficiently induce ubiquitinproteasomal degradation of BRD4 in AR-positive prostate cancer cell lines, with sub-nanomolar half-maximal degradation concentrations (DC_50_) and maximum degradation (*D*_max_) > 99%. Moreover, PROTAC 8 induced cell cycle arrest at the G0/G1 phase and apoptosis in AR-positive prostate cancer by downregulation of the protein levels of AR, PSA, and c-Myc as well as transcriptionally suppressed AR-regulated genes. PROTAC 8 was thus expected to be developed as a promising drug candidate for AR-positive prostate cancer and a valuable tool compound to study the biological function of BRD4.

Targeting CRBN is currently one of the most frequently reported PROTAC approaches, owing to favourable drug-like properties of CRBN ligands, immunomodulatory imide drugs (IMiDs). However, IMiDs are known to be inherently unstable, readily undergoing hydrolysis in body fluids. Min et al. found that IMiD-based PROTACs rapidly hydrolyse in commonly utilised cell media, which significantly affected their cell efficacy. Recently, Rankovic et al. developed novel CRBN binders, phenyl glutarimide analogues, and showed that they retained affinity for CRBN with high ligand efficiency (LE > 0.48) and displayed improved chemical stability. on this basis, they discovered JQ-1-based PROTAC 9 (Figure 4), a uniquely potent degrader of BRD4 that inhibited the viability of human AML MV4-11 cells at low picomolar concentrations (IC_50_=3 pM; BRD4 DC_50_=0.87 nM)^45^. These findings strongly supported the utility of phenyl glutarimide derivatives in the design of CRBN-directed PROTACs.

#### 2.1.2. Targeting BRD2/3/4

**Figure 5.**
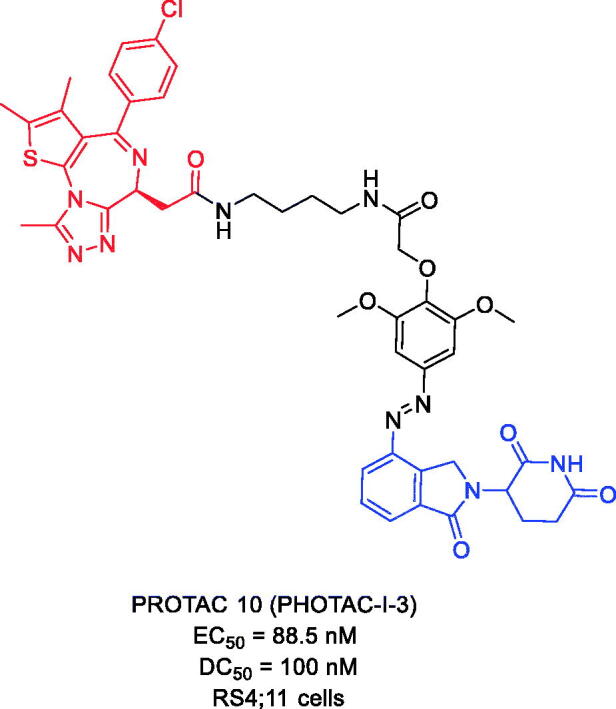
Representative PROTACs targeting BRD2/3/4.

In 2020, Reynders et al. introduced photoswitchable PROTACs that can be activated with the spatiotemporal precision that light provides^46^. These trifunctional molecules, which they named PHOtochemically TArgeting Chimaeras (PHOTACs), consist of a ligand for an E3 ligase, a photoswitch, and a ligand for a POI. They demonstrate this concept by using PHOTACs that target BRD2/3/4 proteins. The representative compound, PROTAC 10 (Figure 5), based on JQ-1, thalidomide derivative, and azobenzenes has been developed. PHOTAC10 showed a promising activity difference upon irradiation. The median effective concentration (EC_50_) was determined to be 88.5 nM when irradiated with 390 nm light and 631 nM in the dark, resulting in a 7.1-fold EC_50_ difference. This indicates that cytotoxicity increases upon irradiation and that PHOTAC 10 is less toxic in the dark. In a control experiment, the BRD inhibitor (+) JQ-1 alone showed no light-dependent toxicity either. Next, they analysed the light dependence of targeted protein degradation in RS4;11 cells by western blot analysis of the BET proteins. To this end, they treated cells with increasing concentrations of our lead compound, PHOTAC 10, for 4 h and pulse irradiated with 390 nm light (100 ms every 10 s). They observed a pronounced decrease in BRD4 levels in the presence of PHOTAC 10 (particularly between 100 nM and 3 M) when irradiated with 390 nm light, but not in the dark. Their modular approach provides a method for the optical control of protein levels with photopharmacology and could lead to new types of precision therapeutics that avoid undesired systemic toxicity.

#### 2.1.3. Targeting BRD9

**Figure 6.**
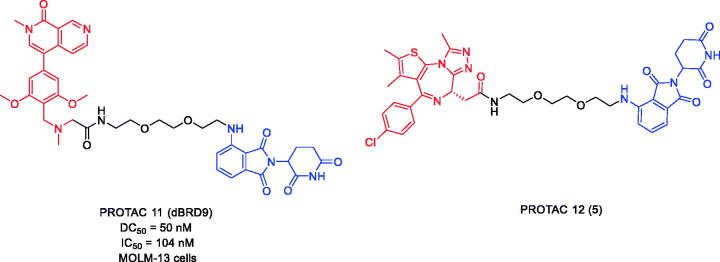
Representative PROTACs targeting BRD9.

BRD9 is a BRD-containing subunit of BAF (BRG-/BRMassociated factor). BAF is a variant of the SWI/SNF complex that regulates gene expression, DNA replication, and DNA repair. Overexpression of BRD9 can lead to cancer development. In 2017, Remillard et al. first developed BRD9 PROTACs by linking BRD9 inhibitor BI-7273 and CRBN ligand pomalidomide^47^. PROTAC 11 (Figure 6) was found to induce degradation of BRD9. It has a significant selectivity for BRD9 over BRD4 and BRD7. Compared to its parental inhibitor BI-7273, PROTAC 11 exhibited 10–100-fold potency in degrading BRD9 with DC_50_ and IC_50_ values of 50 and 104 nM, respectively. The CRBN-based PROTAC targeting BRD9 seems to be a potential strategy for human acute leukaemia treatment.

Despite the growing literature on the synthesis, biological evaluation, and mechanism of action of PROTACs, descriptions of the pharmacokinetic properties of PROTACs are still scarce. In 2020, Goracci et al. reported a study on the metabolism of some BET PROTACs in cryopreserved human hepatocytes at multiple time points^48^. PROTAC 12 (Figure 6) has a combination of BET inhibitor JQ-1 and CRBN ligand pomalidomide attached through a PEG linker. The study showed that the metabolism of PROTAC 12 could not be predicted from the metabolism of their constituent ligands. The chemical nature and length of their linkers played a major role in the responsibility of PROTACs. To interpret the data in more depth, a subset of compounds were also tested for the metabolism of human cytochrome P450 3A4 (CYP3A4) and human aldehyde oxidase (hAOX), both of which lead to active metabolism of PROTACs.

### 2.2. IAP-based PROTACs

#### 2.2.1. Targeting BRD4

**Figure 7.**
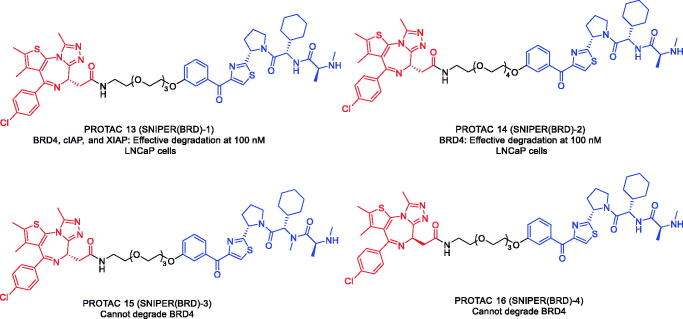
Representative PROTACs targeting BRD4.

In 2019, Ohoka et al. designed and synthesised two BRD4 PROTACs (PROTAC 13 and PROTAC 14, Figure 7) using BRD4 inhibitor JQ-1 and inhibitor of apoptosis proteins (IAP) antagonist LCL-161 derivative^49^. PROTAC 13 and PROTAC 14 could induce marked decreases in BRD4 protein levels at 100 nM. In addition to BRD4, PROTAC 13 also reduced the cIAP1 and XIAP proteins levels within 6 h. The authors found that the degradation of cIAP1 and XIAP by PROTAC 13 was induced by different mechanisms. To study mechanism of action, the authors used a chemical biology-based approach to synthesise PROTAC 15 and PROTAC 16 (Figure 7) that do not degrade BRD4. PROTAC 15 contained an *N*-methylated LCL-161 derivative as the IAP ligand, which prevented it from binding IAP, and resulted in the abrogated degradation of cIAP1, XIAP, and BRD4. PROTAC 16, consisting of the enantiomer (-) JQ-1, was able to degrade cIAP1, but not XIAP and BRD4. In addition, the mixed ligand of JQ-1 and LCL-161 could degrade cIAP1, but not XIAP and BRD4. These results suggested that the degradation of cIAP1 was induced by the binding of the IAP antagonist module triggered by the autoubiquitylation of cIAP1, whereas the PROTAC-induced degradation of XIAP and BRD4 required the formation of ternary complexes.

### 2.3. VHL-based PROTACs

#### 2.3.1. Targeting BRD4

**Figure 8.**
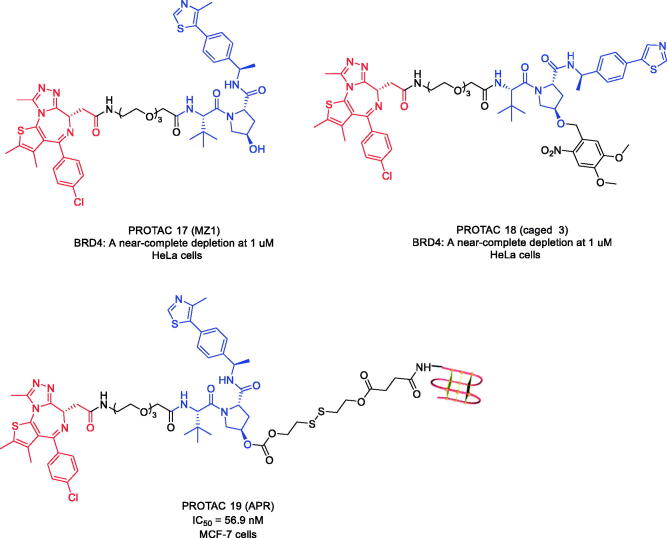
Representative PROTACs targeting BRD4.

Derived from the potent BRD4 inhibitor JQ-1, the first von Hippel-Lindau (VHL)-based PROTAC targeting BRD4 named PROTAC 17 (Figure 8) was reported by Zengerle et al. in 2015^50^. PROTAC 17-induced protein degradation was dependent on binding to VHL E3 ubiquitin ligase. PROTAC 17 showed preferential degradation of BRD4 over BRD2 and BRD3 at low concentrations (When the concentration of PROTAC 17 was 1 μM, more than 90% of BRD4 protein was removed.). Selective depletion of BRD4 with PROTAC 17 produced a different pharmacological response compared to inhibition of the whole BET protein subfamily with JQ-1. Preferential direct interaction or reduced steric constraint between VHL and BRD4 might occur as a result of PROTAC binding, triggering a more productive formation of the VHL-PROTAC 17-BRD4 ternary complex compared to BRD2/3. They concluded that efficient and selective degradation of BRD4 with the PROTAC approach provided an unprecedented opportunity to study the downstream physiological and pathological consequences of BRD4 regulation.

In 2020, Kounde et al. expanded the caged PROTAC toolbox by designing the synthesis of the JQ-1-based PROTAC targeting BRD4 (PROTAC 18, Figure 8) that could be activated on demand with light by attaching a photodegradable 4,5-dimethoxy-2-nitrobenzyl (DMNB) moiety to a VHL ligand^51^. The authors tested the ability of PROTAC 18 to degrade BRD4 after activation with light. HeLa cells were incubated with PROTAC 18 for 2 h before irradiation with a 25 mW 365 nm LED at 80 mm for 60 s. Dose-dependent degradation of BRD4 was observed only at irradiation, with complete knockdown seen at 1 μM. In addition, PROTAC 18 showed good stability in the cellular environment as no degradation was observed in unirradiated cells even after a 24 h incubation period.

The drug development using PROTACs is generally limited by poor membrane permeability, low *in vivo* efficacy and indiscriminate distribution. In 2021, an aptamer-PROTAC conjugation approach was developed as a novel strategy to improve the tumour-specific targeting ability and *in vivo* antitumor potency of conventional PROTACs. As proof of concept, the first aptamer-PROTAC conjugate (APC) was designed by conjugating PROTAC 17 to the nucleic acid aptamer AS1411 (AS) *via* a cleavable linker^52^. Compared with the unmodified PROTAC 17, the designed molecule PROTAC 19 (Figure 8) showed improved tumour targeting ability in a MCF-7 xenograft model, leading to enhanced *in vivo* BET degradation and antitumor potency and decreased toxicity. Thus, the APC strategy may pave the way for the design of tumour-specific targeting PROTACs and have broad applications in the development of PROTAC-based drugs.

#### 2.3.2. Targeting BRD2/3/4

Prostate cancer has the second-highest incidence among cancers in men worldwide and is the second leading cause of cancer deaths of men in the United States. Although androgen deprivation can initially lead to remission, the disease often progresses to castration-resistant prostate cancer (CRPC), which is still reliant on AR signalling and is associated with a poor prognosis. Some success against CRPC has been achieved by drugs that target AR signalling, but secondary resistance invariably emerges, and new therapies are urgently needed. Recently, inhibitors of BET family proteins have shown growth-inhibitory activity in preclinical models of CRPC^53^.

**Figure 9.**
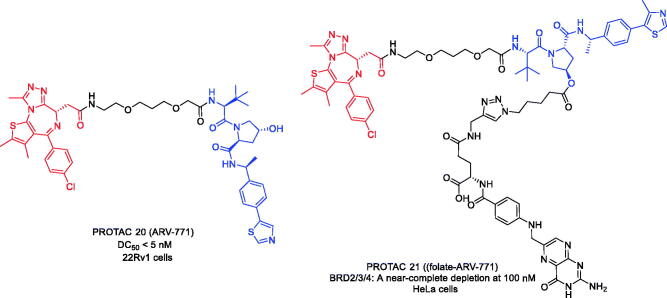
Representative PROTACs targeting BRD2/3/4.

To overcome resistance to second-line anti-androgen therapy (SAT) in patients with CRPC, Raina et al. developed the first BRD2/3/4 PROTAC in 2016^54^. Based on BRD 4 inhibitor JQ-1, PROTAC 20 (Figure 9) was constructed to degrade BRD2/3/4 in the presence of VHL E3 ubiquitin ligase. PROTAC 20 induced the degradation of c-MYC with an IC_50_<1 nM and apoptosis of cells through PARP cleavage. Unlike BET inhibitors, PROTAC 20 was able to suppress both AR signalling and AR levels and led to tumour regression in a CRPC mouse xenograft model. This study demonstrated for the first time the efficacy of small molecule BET degraders in solid tumour malignancies and might represent an important advance in the treatment of CRPC.

In 2021, Liu et al. reported a delivery strategy for FOLR1-targeted PROTAC 20 that selectively degraded BRD2/3/4 proteins in cancer cells *versus* noncancerous normal cells and validated a leading folate- PROTAC 20 (PROTAC 21, Figure 9) that effectively degraded BRD2/3/4 proteins in a FOLR1-dependent manner in cancer cells^55^. However, the conjugation of folate moiety led to an increase in the molecular weight of PROTACs above 1000 Da, which might affect the oral bioavailability and pharmacokinetics of folate-PROTACs. Therefore, more in-depth studies are needed to optimise the stability of PROTAC 21 and to assess its efficiency for cancer-specific delivery *in vivo*.

#### 2.3.3. Targeting BRD2/4

**Figure 10.**
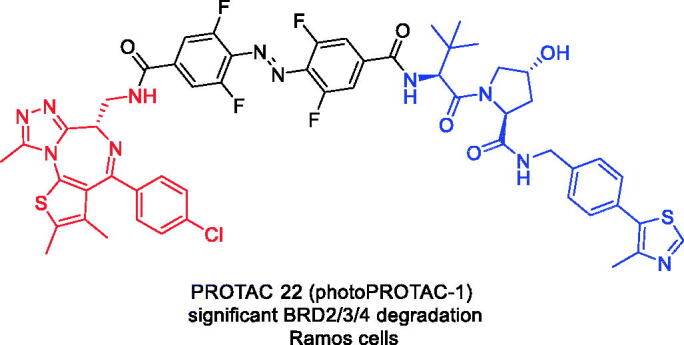
Representative PROTACs targeting BRD2/4.

Although PROTAC 20 is a highly active BET degrader that has been shown to achieve complete regression of prostate cancer in a CRPC mouse xenograft model, it also has general cytotoxic effects. To address the cytotoxic effects of PROTAC 20, Pfaff et al. designed photoswitchable PROTAC (PROTAC 22, Figure 10) by including *ortho*-F4-azobenzene linkers between JQ-1 and VHL ligand^56^. This highly bistable but photoconvertible structural component led to a reversible control of the topological distance between the two ligands. The observed azo-cisisomer was inactive because the distance defined by the linker was too short to allow the formation of a complex between the protein binding partners. In contrast, the azo-trans-isomer was active because it allowed the engagement of both protein partners to form the necessary and productive ternary complex. Importantly, due to the bistable nature of the *ortho*-F4-azobenzene employed, the photostability of PROTAC 22 was durable and did not require continuous irradiation. This technique provided a reversible switch for protein degradation compatible with the intracellular environment and, therefore, might be useful in the experimental exploration of biological signalling pathways, such as those critical for oncogenic signal transduction. By enabling reversible activation and deactivation of protein degradation, PROTAC 22 provided an advantage over conventional photocaging strategies that irreversibly release active agents.

#### 2.3.4. Targeting BRD7/9

**Figure 11.**
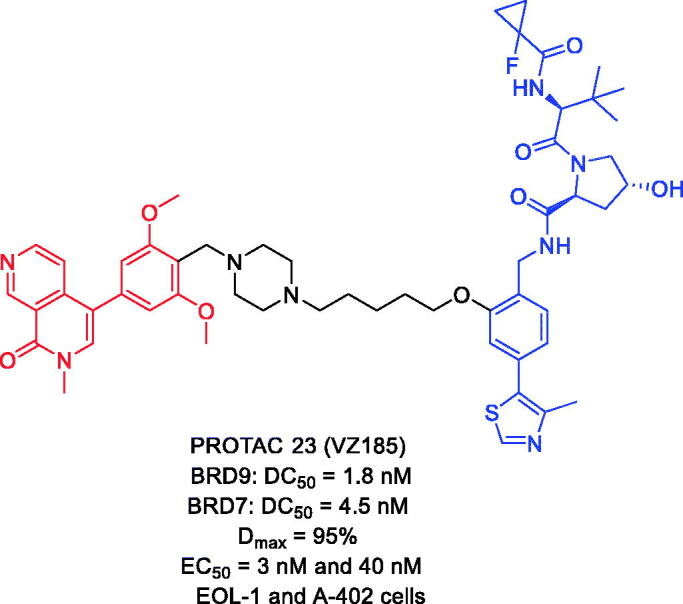
Representative PROTACs targeting BRD7/9.

In 2019, Zoppi et al. discovered PROTAC 23 (Figure 11) as a first-in-class degrader of BRD7 and BRD9. PROTAC 23 was a BI7273-based VHL-recruiting small-molecule degrader of BRD7 and BRD9 with high potency and selectivity^57^. PROTAC 23 could degrade BRD7 and BRD9 with DC_50_ values of 4.5 and 1.8 nM respectively. Two cell lines, acute myeloid eosinophilic leukaemia (EOL-1) and malignant rhabdoid tumour (A-204), sensitive to BRD9 inhibition/degradation and dependent on an active BAF complex, were selected to study the impact of degrader-induced BRD7/9 degradation on the viability of cancer cells. PROTAC 23 showed cytotoxic effects in both cell lines, with EC_50_ values of 3 nM (EOL-1) and 40 nM (A-402), respectively. The authors concluded that PROTACs are a new chemical tool for knocking down BRD7/9.

## 3. Conclusion

BRDs have attracted increasing attention as an important class of targets for drug development, and more than ten BRDs inhibitors have entered early clinical trials targeting patients with different types of cancer. However, BRDs inhibitors have shown very limited antitumor activity in patients, suggesting the need for a new therapeutic approach to target BRDs proteins. Using the PROTACs strategy, highly effective BRDs degraders have now been developed. Preclinical data show that inhibition and degradation of BRDs produce very different cell fates and biological outcomes. BRDs PROTACs may offer a new therapeutic opportunity to target BRDs proteins and greatly improve the limited clinical efficacy of BRDs inhibitors. Compared to BRDs inhibitors, BRDs PROTACs display many features: (1) improving selectivities and specificities; (2) overcoming drug resistances; (3) eliminating the enzymatic and non-enzymatic functions of kinases; (4) rapid and reversible knockdown of POIs. For example, BRDs PROTACs such as dBET1/dBET6, ARV-825/ARV-771, BETd-246/BETd-260, and QCA-570 suppress expression of genes (e.g. c-MYC) regulated by BRDs proteins much more effectively than the corresponding BRDs inhibitors. This has resulted in marked inhibition of cell growth and induction of apoptosis in preclinical models of solid tumours and haematologic malignancies. Glioblastoma cells treated with dBET6 exhibit significant depleted chromatin occupancy of BET proteins, reduced RNA-pol2 activity and impaired transcription program regulated by E2F1. In AML cells, ARV-825 was found to impose a greater perturbation to the mRNA levels than the inhibitor, OTX015.

Although PROTACs are a promising technology, PROTACs still face challenges in the future. First, most of them have high molecular weights, and more practice is needed to assess the absorption, distribution, metabolism, excretion, and toxicity of PROTACs. Second, linkers are also critical for PROTACs. To date, there are no principles guiding the design of linkers. Third, only a few E3 ligases (such as VHL, CRBN, IAP, and MDM2) can be recruited to degrade target proteins within currently chimeric small molecule cells. Although PROTACs face many challenges that need to be addressed, they have the potential to be developed as drugs for the treatment of many incurable diseases.

## References

[1] Drummond ML, Williams CI. In silico modeling of PROTAC-mediated ternary complexes: validation and application. J Chem Inf Model 2019;59:1694–44. 10.1021/acs.jcim.8b00872 30714732

[2] An S, Fu L. Small-molecule PROTACs: an emerging and promising approach for the development of targeted therapy drugs. EBioMedicine 2018;36:553–62. 30224312 10.1016/j.ebiom.2018.09.005 PMC6197674

[3] Wang C, Zhang Y, Wang J, et al. VHL-based PROTACs as potential therapeutic agents: recent progress and perspectives. Eur J Med Chem 2022;227:113906. 34656901 10.1016/j.ejmech.2021.113906

[4] Wang C, Zhang Y, Wu Y, et al. Developments of CRBN-based PROTACs as potential therapeutic agents. Eur J Med Chem 2021;225:113749. 34411892 10.1016/j.ejmech.2021.113749

[5] Farnaby W, Koegl M, Roy MJ, et al. BAF complex vulnerabilities in cancer demonstrated via structure-based PROTAC design. Nat Chem Biol 2019;15:672–80. 31178587 10.1038/s41589-019-0294-6 PMC6600871

[6] Martin-Acosta P, Xiao X. PROTACs to address the challenges facing small molecule inhibitors. Eur J Med Chem 2021;210:112993. 33189436 10.1016/j.ejmech.2020.112993 PMC7779748

[7] Zeng S, Huang W, Zheng X, et al. Proteolysis targeting chimera (PROTAC) in drug discovery paradigm: recent progress and future challenges. Eur J Med Chem 2021;210:112981. 33160761 10.1016/j.ejmech.2020.112981

[8] Smith BE, Wang SL, Jaime-Figueroa S, et al. Differential PROTAC substrate specificity dictated by orientation of recruited E3 ligase. Nat Commun 2019;10:131. 30631068 10.1038/s41467-018-08027-7 PMC6328587

[9] Nowak RP, Deangelo SL, Dennis B, et al. Plasticity in binding confers selectivity in ligand-induced protein degradation. Nat Chem Biol 2018;14:706–14. 29892083 10.1038/s41589-018-0055-y PMC6202246

[10] Wang C, Zhang Y, Xing D, et al. PROTACs technology for targeting non-oncoproteins: advances and perspectives. Bioorg Chem 2021;114:105109. 34175722 10.1016/j.bioorg.2021.105109

[11] Lai AC, Crews CM. Induced protein degradation: an emerging drug discovery paradigm. Nat Rev Drug Discov 2017;16:101–14. 27885283 10.1038/nrd.2016.211 PMC5684876

[12] Gadd MS, Testa A, Lucas X, et al. Structural basis of PROTAC cooperative recognition for selective protein degradation. Nat Chem Biol 2017;13:514–21. 28288108 10.1038/nchembio.2329 PMC5392356

[13] Roy M, Winkler S, Hughes SJ, et al. SPR-measured dissociation kinetics of PROTAC ternary complexes influence target degradation rate. ACS Chem Biol 2019;14:361–8. 30721025 10.1021/acschembio.9b00092 PMC6423499

[14] Barneda-Zahonero B, Parra M. Histone deacetylases and cancer. Mol Oncol 2012;6:579–89. 22963873 10.1016/j.molonc.2012.07.003 PMC5528343

[15] Filippakopoulos P, Picaud S, Mangos M, et al. Histone recognition and large-scale structural analysis of the human bromodomain family. Cell 2012;149:214–31. 22464331 10.1016/j.cell.2012.02.013 PMC3326523

[16] Consortium U. Reorganizing the protein space at the universal protein resource (UniProt). Nucleic Acids Res 2012;40:D71–5. 22102590 10.1093/nar/gkr981 PMC3245120

[17] Belkina AC, Denis GV. BET domain co-regulators in obesity, inflammation and cancer. Nat Rev Cancer 2012;12:465–77. 22722403 10.1038/nrc3256 PMC3934568

[18] Hamm CA, Costa FF. The impact of epigenomics on future drug design and new therapies. Drug Discov Today 2011;16:626–35. 21570477 10.1016/j.drudis.2011.04.007

[19] Dawson MA, Kouzarides T, Huntly BJ. Targeting epigenetic readers in cancer. N Engl J Med 2012;367:647–57. 22894577 10.1056/NEJMra1112635

[20] Dawson MA, Kouzarides T. Cancer epigenetics: from mechanism to therapy. Cell 2012;150:12–27. 22770212 10.1016/j.cell.2012.06.013

[21] Habibi E, Masoudi-Nejad A, Abdolmaleky HM, et al. Emerging roles of epigenetic mechanisms in Parkinson’s disease. Funct Integr Genomics 2011;11:523–37. 21892731 10.1007/s10142-011-0246-z

[22] Pasqualucci L, Dominguez-Sola D, Chiarenza A, et al. Inactivating mutations of acetyltransferase genes in B-cell lymphoma. Nature 2011;471:189–95. 21390126 10.1038/nature09730 PMC3271441

[23] Brownlee PM, Chambers AL, Oliver AW, et al. Cancer and the bromodomains of BAF180. Biochem Soc Trans 2012;40:364–9. 22435813 10.1042/BST20110754

[24] Fujisawa T, Filippakopoulos P. Functions of bromodomain-containing proteins and their roles in homeostasis and cancer. Nat Rev Mol Cell Biol 2017;18:246–62. 28053347 10.1038/nrm.2016.143

[25] Stathis A, Bertoni F. BET proteins as targets for anticancer treatment. Cancer Discov 2018;8:24–36. 201 29263030 10.1158/2159-8290.CD-17-0605

[26] Clark PG, Vieira LC, Tallant C, et al. LP99: discovery and synthesis of the first selective BRD7/9 bromodomain inhibitor. Angew Chem Int Ed Engl 2015;54: 6217–21. 25864491 10.1002/anie.201501394 PMC4449114

[27] Pervaiz M, Mishra P, Günther S. Bromodomain drug discovery - the past, the present, and the future. Chem Rec 2018;18:1808–17. 30289209 10.1002/tcr.201800074

[28] Yang CY, Qin C, Bai L, et al. Small-molecule PROTAC degraders of the Bromodomain and Extra Terminal (BET) proteins - A review. Drug Discov Today Technol 2019;31:43–51. 31200858 10.1016/j.ddtec.2019.04.001

[29] Muddassir M, Soni K, Sangani CB, et al. Bromodomain and BET family proteins as epigenetic targets in cancer therapy: their degradation, present drugs, and possible PROTACs. RSC Adv 2021;11:612–36. 10.1039/d0ra07971e PMC9133982 35746919

[30] Filippakopoulos P, Qi J, Picaud S, et al. Selective inhibition of BET bromodomains. Nature 2010;468:1067–73. 20871596 10.1038/nature09504 PMC3010259

[31] Shi X, Liu C, Liu B, et al. JQ1: a novel potential therapeutic target. Pharmazie 2018;73:491–3. 30223929 10.1691/ph.2018.8480

[32] Leal AS, Williams CR, Royce DB, et al. Bromodomain inhibitors, JQ1 and I-BET 762, as potential therapies for pancreatic cancer. Cancer Lett 2017;394:76–87. 28254412 10.1016/j.canlet.2017.02.021

[33] Doroshow DB, Eder JP, LoRusso PM. BET inhibitors: a novel epigenetic approach. Ann Oncol 2017;28:1776–87. 28838216 10.1093/annonc/mdx157

[34] Shapiro GI, Dowlati A, LoRusso PM, et al. Abstract A49: clinically efficacy of the BET bromodomain inhibitor TEN-010 in an open-label substudy with patients with documented NUT-midline carcinoma (NMC). Mol Cancer Ther 2015;14:A49–A49.

[35] Bui MH, Lin X, Albert DH, et al. Preclinical characterization of BET family bromodomain inhibitor ABBV-075 suggests combination therapeutic strategies. Cancer Res 2017;77:2976–89. 28416490 10.1158/0008-5472.CAN-16-1793

[36] Wang L, Chen Y, Mi Y, et al. ATF2 inhibits ani-tumor effects of BET inhibitor in a negative feedback manner by attenuating ferroptosis. Biochem Biophys Res Commun 2021;558:216–23. 33008584 10.1016/j.bbrc.2020.08.113

[37] Salami J, Crews CM. Waste disposal-An attractive strategy for cancer therapy. Science 2017;355:1163–7. 28302825 10.1126/science.aam7340

[38] Lu J, Qian Y, Altieri M, et al. Hijacking the E3 ubiquitin ligase cereblon to efficiently target BRD4. Chem Biol 2015;22:755–63. 26051217 10.1016/j.chembiol.2015.05.009 PMC4475452

[39] Winter GE, Buckley DL, Paulk J, et al. DRUG DEVELOPMENT. Phthalimide conjugation as a strategy for in vivo target protein degradation. Science 2015;348:1376–81. 25999370 10.1126/science.aab1433 PMC4937790

[40] Bai L, Zhou B, Yang CY, et al. Targeted degradation of BET proteins in triple-negative breast cancer. Cancer Res 2017;77:2476–87. 28209615 10.1158/0008-5472.CAN-16-2622 PMC5413378

[41] Qin C, Hu Y, Zhou B, et al. Discovery of QCA570 as an exceptionally potent and efficacious proteolysis targeting chimera (PROTAC) degrader of the Bromodomain and Extra-Terminal (BET) proteins capable of inducing complete and durable tumor regression. J Med Chem 2018;61:6685–704. 30019901 10.1021/acs.jmedchem.8b00506 PMC6545111

[42] Zhang J, Chen P, Zhu P, et al. Development of small-molecule BRD4 degraders based on pyrrolopyridone derivative. Bioorg Chem 2020;99:103817. 32361153 10.1016/j.bioorg.2020.103817

[43] Bemis TA, La Clair JJ, Burkart MD. Traceless Staudinger ligation enabled parallel synthesis of proteolysis targeting chimera linker variants. Chem Commun (Camb) 2021;57:1026–9. 33406191 10.1039/d0cc05395c PMC7962863

[44] Hu R, Wang WL, Yang YY, et al. Identification of a selective BRD4 PROTAC with potent antiproliferative effects in AR-positive prostate cancer based on a dual BET/PLK1 inhibitor. Eur J Med Chem 2022;227:113922. 34700270 10.1016/j.ejmech.2021.113922

[45] Min J, Mayasundari A, Keramatnia F, et al. Phenyl-glutarimides: alternative cereblon binders for the design of PROTACs. Angew Chem Int Ed Engl 2021;60:26663–70. 34614283 10.1002/anie.202108848 PMC8648984

[46] Reynders M, Matsuura BS, Bérouti M, et al. PHOTACs enable optical control of protein degradation. Sci Adv 2020;6:eaay5064. 32128406 10.1126/sciadv.aay5064 PMC7034999

[47] Remillard D, Buckley DL, Paulk J, et al. Degradation of the BAF complex factor BRD9 by heterobifunctional ligands. Angew Chem Int Ed Engl 2017;56:5738–43. 28418626 10.1002/anie.201611281 PMC5967236

[48] Goracci L, Desantis J, Valeri A, et al. Understanding the metabolism of proteolysis targeting chimeras (PROTACs): the next step toward pharmaceutical applications. J Med Chem 2020;63:11615–38. 33026811 10.1021/acs.jmedchem.0c00793 PMC8015227

[49] Ohoka N, Ujikawa O, Shimokawa K, et al. Different degradation mechanisms of inhibitor of apoptosis proteins (IAPs) by the specific and nongenetic IAP-dependent protein eraser (SNIPER). Chem Pharm Bull (Tokyo) 2019;67:203–9. 30369550 10.1248/cpb.c18-00567

[50] Zengerle M, Chan KH, Ciulli A. Selective small molecule induced degradation of the BET bromodomain protein BRD4. ACS Chem Biol 2015;10:1770–7. 26035625 10.1021/acschembio.5b00216 PMC4548256

[51] Kounde CS, Shchepinova MM, Saunders CN, et al. A caged E3 ligase ligand for PROTAC-mediated protein degradation with light. Chem Commun (Camb) 2020;56:5532–5. 32297626 10.1039/d0cc00523a

[52] He S, Gao F, Ma J, et al. Aptamer-PROTAC conjugates (APCs) for tumor-specific targeting in breast cancer. Angew Chem Int Ed Engl 2021;60:23299–305. 34240523 10.1002/anie.202107347

[53] Wyce A, Degenhardt Y, Bai Y, et al. Inhibition of BET bromodomain proteins as a therapeutic approach in prostate cancer. Oncotarget 2013;4:2419–29. 24293458 10.18632/oncotarget.1572 PMC3926837

[54] Raina K, Lu J, Qian Y, et al. PROTAC-induced BET protein degradation as a therapy for castration-resistant prostate cancer. Proc Natl Acad Sci USA 2016;113:7124–9. 27274052 10.1073/pnas.1521738113 PMC4932933

[55] Liu J, Chen H, Liu Y, et al. Cancer selective target degradation by folate-caged PROTACs. J Am Chem Soc 2021;143:7380–7. 33970635 10.1021/jacs.1c00451 PMC8219215

[56] Pfaff P, Samarasinghe KTG, Crews CM, et al. Reversible spatiotemporal control of induced protein degradation by bistable photoPROTACs. ACS Cent Sci 2019;5:1682–90. 31660436 10.1021/acscentsci.9b00713 PMC6813558

[57] Zoppi V, Hughes SJ, Maniaci C, et al. Iterative design and optimization of initially inactive proteolysis targeting chimeras (PROTACs) identify VZ185 as a potent, fast, and selective von Hippel-Lindau (vhl) based dual degrader probe of BRD9 and BRD7. J Med Chem 2019;62:699–726. 30540463 10.1021/acs.jmedchem.8b01413 PMC6348446

[58] Hugle M, Lucas X, Ostrovskyi D, et al. Beyond the BET family: targeting CBP/p300 with 4-acyl pyrroles. Angew Chem Int Ed Engl 2017;56:12476–80. 28766825 10.1002/anie.201705516

[59] Liu L, Zhen XT, Denton E, et al. ChromoHub: a data hub for navigators of chromatin-mediated signalling. Bioinformatics 2012;28:2205–6. 22718786 10.1093/bioinformatics/bts340 PMC3413389

